# MMP-9-Related microRNAs as Prognostic Markers for Hemorrhagic Transformation in Cardioembolic Stroke Patients

**DOI:** 10.3389/fneur.2019.00945

**Published:** 2019-09-06

**Authors:** Lukai Zheng, Yao Xiong, Junfeng Liu, Xue Yang, Lu Wang, Shuting Zhang, Ming Liu, Deren Wang

**Affiliations:** ^1^Department of Neurology, Center of Cerebrovascular Diseases, West China Hospital of Sichuan University, Chengdu, China; ^2^Department of Neurology, The Third People's Hospital of Chengdu, The Affiliated Hospital of Southwest Jiaotong University, Chengdu, China

**Keywords:** microRNA, hemorrhagic transformation, cardioembolic stroke, MMP-9, prediction

## Abstract

Studies suggest that microRNAs that regulate expression of matrix metalloproteinase (MMP)-9 may be involved in hemorrhagic transformation (HT) after cardioembolic stroke, so we examined whether such microRNAs could predict HT in acute cardioembolic stroke patients. Blood samples were prospectively collected from patients who later experienced HT (*n* = 29) or did not (*n* = 29), and the samples were assayed for eight microRNAs identified as related to MMP-9 based on three microRNA databases. Expression levels of these microRNAs were analyzed by quantitative real-time polymerase chain reaction (qRT-PCR) in 28 of the 58 patients, 14 of whom suffered HT and 14 of whom did not. Four differentially expressed miRNAs were identified: *hsa-miR-21-5p, hsa-miR-206, hsa-miR-491-5p*, and *hsa-miR-3123*. Subsequent qRT-PCR analysis of these four miRNAs across all 58 patients showed that levels of *miR-21-5p, miR-206*, and *miR-3123* were significantly higher in patients with HT than in those without HT, while expression of *miR-491-5p* was similar between the two groups. The area under the receiver operating characteristic curve for predicting HT was 0.677 (95% CI 0.535–0.818) for *miR-21-5p*, 0.687 (95% CI 0.543–0.830) for *miR-206*, and 0.661 (95% CI 0.512–0.810) for *miR-3123*. Our results suggest that these three microRNAs may be prognostic markers for HT after cardioembolic stroke, which should be verified by future studies with large samples.

## Introduction

Hemorrhagic transformation (HT) occurs in some ischemic stroke patients. HT can occur naturally following ischemic infarction or be triggered by therapeutic interventions (1, 2). The reported incidence of spontaneous HT in Western countries ranges from 10 to 40% and in the Chinese population from 23 to 43% (3–6). Cardioembolic strokes, such as those caused by atrial fibrillation, are associated with an increased risk of HT and their prognosis could be worsened by HT (7, 8). The fear of HT and its associated complications hinder treatments for cardioembolic stroke, including reperfusion therapy, early anticoagulation, and dual-antiplatelet agents (9). Although thrombolysis for acute stroke and anticoagulation for stroke patients with atrial fibrillation are recommended by national health guidelines in many countries including China (10, 11), only 2–5% of Chinese patients with acute ischemic stroke receive thrombolysis (12, 13). Among patients who do not receive thrombolysis, the reason in more than one third of cases is concern over the risk of HT (14). Similarly, only 21–37% of stroke patients with atrial fibrillation receive anticoagulation in China (15). About 32% of stroke patients with atrial fibrillation do not receive anticoagulation because of concerns about the risk of intracranial hemorrhage (16). Therefore, finding predictors for identifying patients that are at high risk of HT would improve treatment of cardioembolic stroke.

Matrix metalloproteinase-9 (MMP-9) participates in multiple physiological and pathological processes in the central nervous system. MMP-9 is an enzyme that digests type IV collagen, laminin, and fibronectin, which are major components of the basal lamina in cerebral blood vessels (17). Animal and human studies have demonstrated that increased MMP-9 expression plays an important role in blood-brain barrier (BBB) leakage and is significantly increased in patients with HT after ischemic stroke (18). Systematic review of previous studies concluded that MMP-9 independently predicts HT in acute stroke patients after the use of recombinant tissue plasminogen activator (19). These studies collectively demonstrated that MMP-9 may be involved in HT after acute ischemic stroke, possibly through mechanisms related to BBB disruption.

MicroRNAs (miRNAs) are small, non-coding RNA molecules of 20–24 nucleotides that regulate gene expression at the posttranscriptional level (20). Certain miRNAs regulate MMP-9 expression during stroke (21). Furthermore, some miRNAs that are highly expressed in brain tissue can similarly be detected in peripheral blood (22). These results suggest that circulating miRNAs may serve as clinical biomarkers for stroke.

In the current study, we hypothesized that some miRNAs that are related to MMP-9 would be prognostic biomarkers for predicting HT following cardioembolic stroke. Therefore, we tested the potential association between these miRNAs and HT after cardioembolic stroke in a patient sample.

## Materials and Methods

### Patient Selection

A total of 58 cardioembolic stroke patients admitted within 7 days of stroke onset were randomly selected from the Department of Neurology, West China Hospital of Sichuan University (Chengdu, China) between September 2013 and January 2017. Among these patients, 29 patients (66 ± 13 years old; 72.4% female) later experienced HT and the other 29 patients (67 ± 14 years old; 79.3% female) did not. No significant differences were found between two groups regarding age, gender, risk factors, and anti-thrombotic medications used before admission. The datasets supporting the findings of the current study are available from the corresponding author upon reasonable request.

All patients received a clinical diagnosis of stroke according to World Health Organization criteria (23), and this diagnosis was confirmed by computed tomography (CT) or magnetic resonance imaging (MRI). Cardioembolic etiology was identified according to the Trial of ORG 10172 in Acute Stroke Treatment (TOAST) classification (24). Only Han Chinese adults over 18 years of age were included in this study. Patients were excluded if they (1) had intracerebral hemorrhage (ICH) before or at admission; (2) had recent head injuries, hematological disorders, severe renal or liver dysfunction, malignancies, systemic infection, or large-artery stenosis due to atherosclerosis or arteritis; (3) received immunosuppressive therapies before or during admission; (4) received thrombolytic therapies after stroke onset; or (5) were unable to take care of themselves for any reason prior to stroke onset.

### Baseline Data Collection

We recorded baseline clinical and demographic information, including age; sex; ethnicity; stroke severity assessed by the National Institutes of Health Stroke Scale (NIHSS) on admission (25); pre-existing risk factors, including history of hypertension, diabetes mellitus, hyperlipidemia, atrial fibrillation, coronary artery disease, ICH, ischemic stroke or transient ischemic attack, current smoking, and alcohol consumption; anti-thrombotic medications used before admission, such as antiplatelet agents and anticoagulants; date of stroke symptom onset; and time of blood sampling and neuroimaging. All patients in our study underwent CT within 24 h on arrival at the emergency department, which is the point of entry to our comprehensive stroke center at West China Hospital (26). Patients received another CT or MRI again between 2 days and 2 weeks after stroke onset or within 2 days after worsening of the patient's condition. Any changes in symptoms or signs were recorded. CT and MRI were evaluated by a neuroradiologist blinded to patient details, including clinical characteristics of the stroke. HT was identified when hemorrhage within the acute ischemic lesion was not detected by the initial CT but was later confirmed by a second CT or MRI (27).

### Blood Sampling

Venipuncture was prospectively performed in all inpatients within 24 h after arrival in the emergency department, when none of the 58 patients was known to have HT. Venous blood was collected in EDTA-containing tubes and was then sent to the laboratory within 30 min of collection. After centrifugation (3,000 rpm for 15 min at 4°C), plasma was collected and immediately frozen at −80°C until further use.

### Selection and Assessment of Candidate MMP-9-related MiRNAs

TargetScan version 6.2 (www.targetscan.org), miRanda August 2010 release (www.microrna.org/microrna/home.do) and miRDB version 4.0 (www.mirdb.org) were used to predict miRNAs acting upstream of the human *MMP9* gene *in silico*. A literature review was also performed in PubMed to identify candidate miRNA regulators of MMP-9 expression.

### Extraction of miRNAs, Reverse Transcription and qRT-PCR

Plasma fractions enriched for miRNAs were isolated from plasma samples using the miRcute miRNA Isolation Kit (Tiangen, Beijing, China). Before this isolation step, all plasma samples were spiked with *ath-miR156a* (2.5 μl, 1 μM), which shows no sequence homology to human miRNAs and therefore served as a reference RNA. Fractions enriched in miRNA were eluted in 30 μl, and a 3 μl aliquot was treated with gDNA Eraser (Takara, Japan) to eliminate potential contaminating genomic DNA, then reverse-transcribed using the PrimeScript RT reagent Kit (Takara). Sangon (Shanghai, China) designed miRNA-specific stem-loop primers for reverse transcription (Supplementary Table).

Quantitative real-time polymerase chain reaction (qRT-PCR) was performed using a C1000 Touch Thermal Cycler (CFX96 Real-Time System, Bio-Rad, Hercules, CA, USA) with SsoFast EvaGreen Surpermix (Bio-Rad). Forward and reverse primers for each miRNA in qRT-PCR were obtained from Sangon (Supplementary Table). The resulting solubility curve and cycle threshold (Ct) were evaluated via CFX Manager software (Bio-Rad), and relative expression (fold difference) of candidate miRNAs with respect to the average level in patients who did not suffer HT (NHT) was calculated using the **2^−ΔΔCt^ method according to the formula: fold difference = 2^−[(Ct Candidate−Ct miR156a)−(Mean Ct Candidate_NHT_−Mean Ct miR156a_NHT_)]^**.

### Statistical Analyses

Categorical variables were reported as percentages. Continuous variables were reported as mean ± standard deviation or median (interquartile range [IQR]). Data normality was examined using the Shapiro–Wilk test. Results for categorical variables were compared between groups using Fisher exact tests. Results for continuous variables were compared using the Welch *t*-tests or Mann–Whitney *U*-tests. Differences were considered significant when *p* < 0.05 (two-tailed). All statistical analyses were performed using R version 3.4.4 (www.R-project.org).

Receiver operating characteristic (ROC) curves were constructed to assess the sensitivity and specificity of MMP-9-related miRNAs for predicting HT after cardioembolic stroke. The prognostic values of specific miRNAs were assessed based on the area under the curve (AUC). For further analyses, partial AUCs (pAUCs) were generated for the specificity range of 80–100%, meaning that a negative blood test should correctly identify at least 8 of 10 patients as having low risk of HT.

## Results

A three-step approach was adopted to identify whether MMP-9-related miRNAs were prognostic biomarkers for predicting HT following cardioembolic stroke. First, only the miRNA-mRNA pairs simultaneously predicted by at least two of three databases (see Methods) were considered as possible miRNA-mRNA pairs (Supplementary Figure). This step identified x candidate miRNAs upstream of the *MMP9* gene: *hsa-miR-183-5p, hsa-miR-204-5p, hsa-miR-206, hsa-miR-491-5p, hsa-miR-211-5p, hsa-miR-3123*, and *hsa-miR-3145-5p*. We also included *hsa-miR-21-5p*, a well-known miRNA that has been linked to MMP-9 (28).

**Figure 1:**
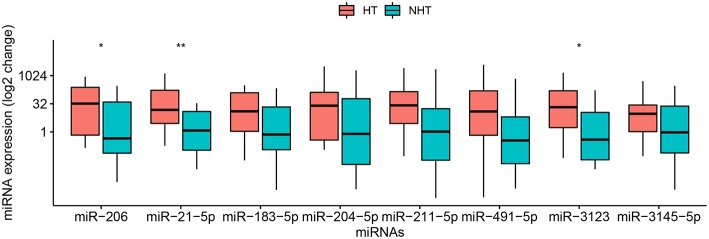
Relative levels of 8 miRNAs in plasma of 28 patients with cardioembolic stroke. Patients were stratified into 14 who experienced hemorrhagic transformation (HT) and 14 who did not (NHT). Relative miRNA levels were measured by qRT-PCR and calculated using the 2^−ΔΔCt^ method. Results are presented in the log_2_ scale in the boxplot, where the upper limit of the box is 75th percentile, lower limit is 25th percentile, and the lines inside the box indicate the median. The Mann–Whitney *U*-test was used for statistical analysis. *p < 0.05; **p < 0.01.

Second, a pilot study was performed to screen levels of the 8 candidate miRNAs in 28 of the 58 patients, comprising 14 who suffered HT and 14 who did not (Table 1). This study showed that levels of circulating *miR-21-5p* (*p* = 0.009), *miR-206* (*p* = 0.037), and *miR-3123* (*p* = 0.036) were significantly higher in HT patients (Figure 1). In contrast, levels of *miR-491-5p, miR-183-5p, miR-211-5p, miR-3145-5p*, and *miR-204-5p* did not differ significantly between the two patient groups. The remaining miRNA, *miR-491-5p*, showed a trend toward statistical significance between groups: the median 2^−ΔΔCt^ value was 18.38 (0.66–161.49) in HT vs. 0.35 (0.02–10.12) without HT (*p* = 0.0594). Therefore, it was included in further investigation.

**Table 1:** Demographic and clinical characteristics of patients with cardioembolic stroke used to test miRNAs predicted *in silico* to affect MMP-9 expression.

**Characteristic**	**HT (*n* = 14)**	**No HT (*n* = 14)**	p*
Age, mean ± SD, year	66 ± 13	65 ± 15	0.907
Female, *n* (%)	9 (64.3)	11 (78.6)	0.678
Onset to blood sampling, h	12 (4–25)	28 (8–76)	0.164
Onset to baseline CT or MRI, h	23 (7–31)	27 (9–77)	0.454
Onset to admission, h	24 (20–67)	35 (26–90)	0.334
NIHSS on admission	10 (5–17)	3 (1–7)	**0.025**
Systolic blood pressure, mean ± SD, mmHg	131 ± 24	127 ± 29	0.687
Diastolic blood pressure, mean ± SD, mmHg	81 ± 18	79 ± 13	0.700
Risk factors, *n* (%)			
Hypertension	8 (57.1)	3 (21.4)	0.120
Diabetes mellitus	2 (14.3)	0 (0)	0.482
Hyperlipidemia	1 (7.1)	1 (7.1)	1.000
Coronary heart disease	2 (14.3)	1 (7.1)	1.000
Atrial fibrillation	11 (78.6)	10 (71.4)	1.000
Current smoking	3 (21.4)	0 (0)	0.222
Alcohol consumption	1 (7.1)	0 (0)	1.000
History of stroke/TIA	2 (14.3)	3 (21.4)	1.000
Antiplatelet therapy before admission	2 (14.3)	1 (7.1)	1.000
Anticoagulation therapy before admission	5 (35.7)	5 (35.7)	1.000

*Values are n (%) for categorical variables and median (interquartile range) for continuous variables, unless otherwise indicated*.

CT, computed tomography; HT, hemorrhagic transformation; MRI, magnetic resonance imaging; NIHSS, National Institutes of Health Stroke Scale; SD, standard deviation; TIA, transient ischemic attack.

* *p calculated for the Welch t-test for age and blood pressure, for the Mann–Whitney U-test for other numeric variables, or for the Fisher exact test for categorical variables. Significance is indicated by bold marking when p < 0.05 (two-tailed p-value)*.

**Figure 2:**
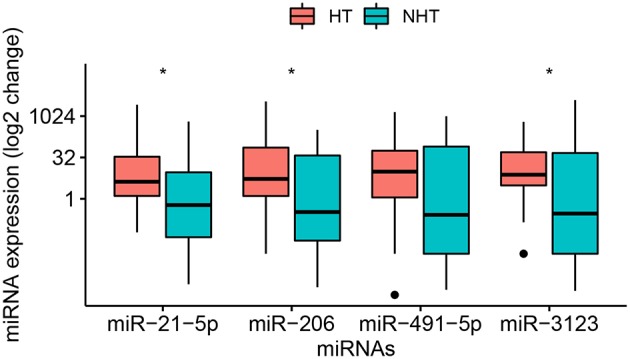
Relative levels of *miR-21-5p, -206, -491-5p*, and *-3123* in plasma of the entire sample of 58 patients with cardioembolic stroke. Data were obtained and displayed as described in Figure 1, except that points indicate values falling outside 80% of the data. The Mann–Whitney *U*-test was used for statistical analysis. *p < 0.05.

Third, the three miRNAs that were significantly different between HT and non-HT patients, along with *miR-491-5p*, were analyzed in all 58 patients, whose baseline characteristics are shown in Table 2. Half these patients developed HT subsequently. The median age for all 58 patients was 70.5 (55.3–78.0) years, and the interval between onset and hospitalization was 27 (12–72) h. The interval between stroke onset and blood collection was 16 (6–48) h. Among the characteristics that we assessed, only NIHSS score on admission differed significantly between patients who developed HT or not: the score was higher among those with HT [11 (6–14) vs. 4 (3–14), *p* = 0.015]. Of the four candidate miRNA markers, three were expressed at significantly higher levels among patients with HT than among those without HT (Figure 2). The comparisons of 2^−ΔΔCt^ values between the two groups were as follows: *miR-21-5p*, 4.17 (1.27–33.95) vs. 0.59 (0.04–9.21) (*p* = 0.021); *miR-206*, 5.29 (1.42–74.26) vs. 0.33 (0.03–37.66) (*p* = 0.016); and *miR-3123*, 7.54 (3.07–49.62) vs. 0.29 (0.01–46.50) (*p* = 0.036). In contrast, *miR-491-5p* level was similar between patients who suffered HT or not [9.74 (1.12–56.27) vs. 0.26 (0.01–79.72), *p* = 0.109].

**Table 2:** Demographic and clinical characteristics of all 58 patients used to validate the candidate miRNAs predicted *in silico* to regulate MMP-9 expression*.

**Characteristic**	**HT (*n* = 29)**	**No HT (*n* = 29)**	p**
Age, year	66 ± 13	67 ± 14	0.762
Female, *n* (%)	21 (72.4)	23 (79.3)	0.760
Onset to blood sampling, h	13 (6–46)	27 (7–50)	0.375
Onset to baseline CT or MRI, h	14 (6–51)	26 (7–73)	0.478
Onset to admission, h	24 (12–72)	40 (18–72)	0.691
NIHSS on admission	11 (6–14)	4 (3–14)	**0.015**
Systolic blood pressure, mean ± SD, mmHg	131 ± 25	133 ± 24	0.852
Diastolic blood pressure, mean ± SD, mmHg	82 ± 17	81 ± 12	0.860
Risk factors, *n* (%)			
Hypertension	14 (48.3)	8 (27.6)	0.176
Diabetes mellitus	4 (13.8)	2 (6.9)	0.670
Hyperlipidemia	1 (3.5)	2 (6.9)	1.000
Coronary heart disease	3 (10.3)	3 (10.3)	1.000
Atrial fibrillation	20 (69.0)	17 (58.6)	0.585
Current smoking	4 (13.8)	3 (10.3)	1.000
Alcohol consumption	4 (13.8)	3 (10.3)	1.000
History of stroke/TIA	4 (13.8)	6 (20.7)	0.730
Antiplatelet therapy before admission	3 (10.3)	4 (13.8)	1.000
Anticoagulation therapy before admission	6 (20.7)	7 (24.1)	1.000

*Values are n (%) for categorical variables and median (interquartile range) for continuous variables, unless otherwise indicated*.

*CT, computed tomography; HT, hemorrhagic transformation; MRI, magnetic resonance imaging; NIHSS, National Institutes of Health Stroke Scale; SD, standard deviation; TIA, transient ischemic attack*.

* *These patients include the 28 patients used to assess the initial set of miRNAs identified in silico (Table 1)*.

** *p calculated by Welch t-test for age and blood pressure, by Mann–Whitney U-test for other numeric variables, by Fisher exact test for categorical variables. Significance is indicated by bold marking when p < 0.05 (two-tailed p-value)*.

**Figure 3:**
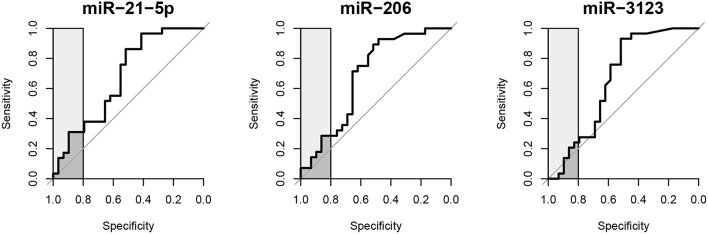
Receiver operating characteristic curves of *miR-21-5p, miR-206*, and *miR-3123* for predicting HT following cardioembolic stroke. Light gray boxes highlight the top 20% of specificity. Dark gray boxes correspond to the partial area under the curve.

The prognostic value of circulating *miR-21-5p, miR-206*, and *miR-3123* for HT after cardioembolic stroke was then assessed using ROC curves and the pROC package in R (29). The AUC of *miR-21-5p* was 0.677 (95% CI 0.535–0.818); *miR-206*, 0.687 (95% CI 0.543–0.830); *miR-3123*, 0.661 (95% CI 0.512–0.810). Threshold values for predicting HT in the 58 patients were defined based on the upper 80% of the ROC curves (Figure 3 and Table 3). The *miR-21-5p* showed 31.0% sensitivity and 89.7% specificity with a threshold above a 2^−ΔΔCt^ value of 26.90, and *miR-206* and *miR-3123* also showed low respective sensitivities of 28.6 and 24.1%. The pAUC for *miR-21-5p* was 0.042 (95% CI 0.013–0.080); *miR-206*, 0.034 (95% CI 0.007–0.076); and *miR-3123*, 0.020 (95% CI 0–0.059).

**Table 3:** Partial AUCs, sensitivities and specificities for *miR-21-5p, miR-206*, and *miR-3123* in receiver operating characteristic analysis.

**miRNA**	Partial AUC* (%) (95%CI)	Threshold (2^**−ΔΔCt**^ value**)	**Sensitivity (%) (95%CI)**	**Specificity (%) (95%CI)**
*miR-206*	3.4 (0.8–7.4)	64.96	28.6 (14.3–46.3)	86.2 (72.4–96.6)
*miR-21-5p*	4.2 (1.3–8.0)	26.90	31.0 (17.2–48.3)	89.7 (75.9–100)
*miR-3123*	2.0 (0–5.9)	51.46	24.1 (10.3–41.4)	82.8 (69.0–96.6)

*AUC, area under the curve. CI, confidence interval*.

* *Partial AUCs are restricted to specificity ranging from 80 to 100%*.

** *Relative miRNA expression was normalized with the reference miRNA ath-miR156a and quantified using the 2^−ΔΔCt^ method*.

## Discussion

In this case-control study, analysis by qRT-PCR suggested that expression of *miR-21-5p, miR-206*, and *miR-3123* in plasma increases before HT occurs in cardioembolic stroke patients. Consistent with this idea, we found preliminary evidence that plasma levels of *miR-21-5p, miR-206*, and *miR-3123* can predict HT after cardioembolic stroke.

Following acute ischemic stroke, there are numerous pathological processes that can cause BBB disruptions and theoretically increase the risk of HT, including apoptosis, changes in metabolism, and neuro-inflammation. Mounting evidence indicates that some miRNAs participate in these processes, making them potential markers of brain tissue injury after stroke onset (30). Compared with RNA molecules, circulating miRNAs are relatively stable in serum or plasma because they are enclosed within acid- and base-resistant membrane vesicles (31), allowing their quantitation by qRT-PCR.

Existing evidence shows that *miR-21-5p*, an miRNA highly expressed in vascular smooth muscle and endothelial cells (32), is related to stroke and cardiovascular disease (33). Serum expression levels of *miR-21-5p* have been found to correlate positively with serum activity of MMP-9 in acute coronary syndrome (34). *In vivo* ischemia/reperfusion injury models showed that *miR-21-5p* reduces cell apoptosis and promotes angiogenesis, thereby protecting tissue from damage (32, 35). A protective role for *miR-21-5p* is consistent with our observation of elevated levels of this miRNA in our group of patients with HT, who showed higher (worse) NIHSS scores than patients without HT. Interestingly, *miR-21-5p* did not emerge from our *in silico* screening. Nevertheless, we chose to include it because MMP-9 is regulated at stages other than transcription (28, 36), and it may regulate two MMP inhibitors (32, 37).

A skeletal muscle-specific member of the MyomiR family, *miR-206* has an important role in myogenic differentiation (38). Evidence from an *in vivo* study indicates that *miR-206* inhibits tissue inhibitor of MMP-3 and increases levels of MMP-9 and inflammatory factors (39). Moreover, levels of *miR-206* positively correlated with infarct volume in an animal model of embolic stroke (40). These findings, together with our results, suggest that elevated expression of *miR-206* after cardioembolic stroke exacerbates neuroinflammation and ischemic damage, thereby justifying further assessment of *miR-206* as a strong candidate prognostic marker for HT after stroke.

We further identified a link between *miR-3123* and risk of HT in patients with cardioembolic stroke. Although experiments have suggested no relationship between *miR-3123* levels and MMP-9 expression (36), our *in silico* tests predict it as a regulator of this enzyme. Future studies should explore this possibility further.

The prognostic value of *miR-21-5p, -206* or *-3123* on their own appears to be limited, based on pAUC values, which are restricted to specificity of 80–100%. Nevertheless, if a cardioembolic stroke patient shows high values of any one of these, it may be appropriate to consider him or her at high risk of HT. Future studies should examine whether combinations of these or other miRNAs can provide a more sensitive and specific marker of risk.

In the clinical setting, one potential limitation of qRT-PCR is that it may require longer than is available for timely treatment after stroke onset. Median interval from alteplase infusion to symptomatic ICH was reported to range from 5 to 10 h (2), and the qRT-PCR in our study took more than 5 h. Intravenous alteplase therapy, if indicated, should not be delayed to await the results of plasma miRNA testing (10). For this reason, we excluded from our study patients receiving thrombolytic therapies after stroke onset.

There are several limitations in our study. This was a case-control study based on small numbers of samples in one hospital. Blood samples were not collected longitudinally, which may be important for understanding changes in miRNA expression after stroke onset. In addition to further investigations of the three miRNAs' role in MMP-9 regulation, neurological deterioration and HT development, future studies should perform longitudinal analysis of a large, preferably multi-center population to develop a complete, time-specific miRNA expression profile for HT prediction in cardioembolic stroke patients. Regardless, our study provides preliminary and intriguing hypothesis-generating findings to lead the way for future advancement in the relationship between miRNA and HT.

## Data Availability

The datasets generated for this study are available on request to the corresponding author.

## Ethics Statement

This study was reviewed and approved under the ethical project identification code 2015(300) by the Biomedical Ethics Committee of West China Hospital, Sichuan University. Written informed consent was obtained from all patients or their guardians.

## Author Contributions

DW and ML conceived the project and designed the study. LZ, YX, XY, and LW recruited patients and acquired clinical data. YX, LZ, JL, and SZ performed the biomarker experiments, then collected, and analyzed data. LZ and YX wrote the manuscript, which DW revised. All authors have read, revised, and approved the final version of the manuscript.

### Conflict of Interest Statement

The authors declare that the research was conducted in the absence of any commercial or financial relationships that could be construed as a potential conflict of interest.
