# Supplementary material for: MMP-9-Related microRNAs as Prognostic Markers for Hemorrhagic Transformation in Cardioembolic Stroke Patients
Source: Front Neurol. 2019 Sep 6;10:945. doi: 10.3389/fneur.2019.00945 (PMC6742920; doi:10.3389/fneur.2019.00945)
Supplement: Supplementary file 1 [file Data_Sheet_1.PDF]

Supplementary Material

Supplementary Table. Sequences of RT primers and forward primers for qRT-PCR. A universal reverse primer (5'-ATCCAGTGCAGGGTCCGAGG-3') was used for all the microRNAs in qRT-PCR and are therefore not listed in the table.

microRNA	Primer sequence (5'-3')
cel-miR-39-3p	RT GTCGTATCCAGTGCAGGGTCCGAGGTATTCGCACTGGATACGACCAAGCT
	Forward ATTGCGGTCACCGGGTGTAAATC
hsa-miR-206	RT GTCGTATCCAGTGCAGGGTCCGAGGTATTCGCACTGGATACGACCCACAC
	Forward CGGCGGTGGAATGTAAGGAAGT
hsa-miR-21-5p	RT GTCGTATCCAGTGCAGGGTCCGAGGTATTCGCACTGGATACGACTCAACA
	Forward CGGCGGTAGCTTATCAGACTGA
hsa-miR-183-5p	RT GTCGTATCCAGTGCAGGGTCCGAGGTATTCGCACTGGATACGACAGTGAA
	Forward TTGGCGGTATGGCACTGGTAGAA
hsa-miR-204-5p	RT GTCGTATCCAGTGCAGGGTCCGAGGTATTCGCACTGGATACGACAGGCAT
	Forward AAGGCGGTTCCCTTTGTCATCCT
hsa-miR-211-5p	RT GTCGTATCCAGTGCAGGGTCCGAGGTATTCGCACTGGATACGACAGGCGA
	Forward AAGGCGGTTCCCTTTGTCATCCT
hsa-miR-491-5p	RT GTCGTATCCAGTGCAGGGTCCGAGGTATTCGCACTGGATACGACCCTCAT
	Forward TTATTGGAGTGGGGAACCCTTCC
hsa-miR-3123	RT GTCGTATCCAGTGCAGGGTCCGAGGTATTCGCACTGGATACGACGATTAA
	Forward TATTGGCGGCGGCAGAGAATTGT
hsa-miR-3145-5p	RT GTCGTATCCAGTGCAGGGTCCGAGGTATTCGCACTGGATACGACTGAGTT
	Forward TCGGCGGAACTCCAAACACTCAA

RT, reverse transcription; qRT-PCR, quantitative real-time polymerase chain reaction.

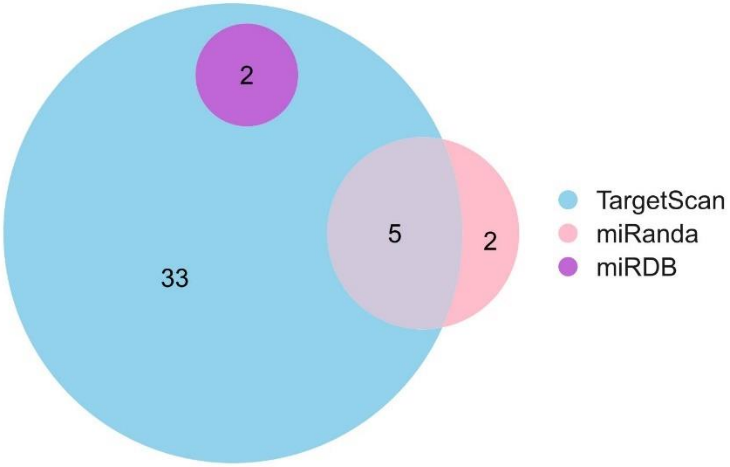

Supplementary Figure. Venn diagram showing overlaps among miRNAs predicted to be related to MMP-9 by TargetScan, miRanda and miRDB. Two miRNAs (*hsa-miR-3123* and *hsa-miR-3145-5p*) were simultaneously predicted by TargetScan and miRDB, while five miRNAs (*hsa-miR-183-5p*, *hsa-miR-204-5p*, *hsa-miR-206*, *hsa-miR-211-5p* and *hsa-miR-491-5p*) were simultaneously predicted by TargetScan and miRanda.
